# Assessment of the Risk of Medium-Term Internal Contamination in Minamisoma City, Fukushima, Japan, after the Fukushima Dai-ichi Nuclear Accident

**DOI:** 10.1289/ehp.1306848

**Published:** 2014-03-14

**Authors:** Amina Sugimoto, Stuart Gilmour, Masaharu Tsubokura, Shuhei Nomura, Masahiro Kami, Tomoyoshi Oikawa, Yukio Kanazawa, Kenji Shibuya

**Affiliations:** 1 Department of Global Health Policy, Graduate School of Medicine, The University of Tokyo, Tokyo, Japan; 2 Division of Social Communication System for Advanced Clinical Research, The Institute of Medical Science, The University of Tokyo, Tokyo, Japan; 3 Department of Neurosurgery, Minamisoma Municipal General Hospital, Fukushima, Japan

## Abstract

Background: The Fukushima Dai-ichi nuclear disaster, the first level-7 major nuclear disaster since Chernobyl, raised concerns about the future health consequences of exposure to and intake of radionuclides. Factors determining the risk and level of internal radiation contamination after a nuclear accident, which are a key to understanding and improving current nuclear disaster management, are not well studied.

Objective: We investigated both the prevalence and level of internal contamination in residents of Minamisoma, and identified factors determining the risk and levels of contamination.

Methods: We implemented a program assessing internal radiation contamination using a whole body counter (WBC) measurement and a questionnaire survey in Minamisoma, between October 2011 and March 2012.

Results: Approximately 20% of the city’s population (8,829 individuals) participated in the WBC measurement for internal contamination, of which 94% responded to the questionnaire. The proportion of participants with detectable internal contamination was 40% in adults and 9% in children. The level of internal contamination ranged from 2.3 to 196.5 Bq/kg (median, 11.3 Bq/kg). Tobit regression analysis identified two main risk factors: more time spent outdoors, and intake of potentially contaminated foods and water.

Conclusions: Our findings suggest that, with sensible and reasonable precautions, people may be able to live continuously in radiation-affected areas with limited contamination risk. To enable this, nuclear disaster response should strictly enforce food and water controls and disseminate evidence-based and up-to-date information about avoidable contamination risks.

Citation: Sugimoto A, Gilmour S, Tsubokura M, Nomura S, Kami M, Oikawa T, Kanazawa Y, Shibuya K. 2014. Assessment of the risk of medium-term internal contamination in Minamisoma City, Fukushima, Japan, after the Fukushima Dai-ichi Nuclear accident. Environ Health Perspect 122:587–593; http://dx.doi.org/10.1289/ehp.1306848

## Introduction

On 11 March 2011, the Fukushima Dai-ichi nuclear power plant in northeast Japan was substantially damaged by the Great East Japan Earthquake and its subsequent tsunami [World Health Organization (WHO) 2013]. Approximately 900 PBq of radionuclides, including 500 PBq of iodine-131 (^131^I) and a total of 20 PBq cesium isotopes (^134^Cs and ^137^Cs), were released into the atmosphere, contaminating > 1,800 km^2^ of land [The National Diet of Japan Fukushima Nuclear Accident Independent Investigation Commission (NAIIC) 2012; Tokyo Electronic Power Company 2012]. Although the amount of radionuclides released was just one-sixth of that from the Chernobyl accident (Taira et al. 2013), people in areas affected by the disaster received annual effective doses up to 5 mSv, which was equivalent to the standard that was fixed 5 years after the Chernobyl accident for recommended relocation (Nuclear Energy Agency 2002; Taira et al. 2013; WHO 2013). Accordingly, the disaster has raised concerns about the short- and long-term health consequences of radiation in Japan and worldwide.

Confronted with the disaster, the Japanese government immediately mandated several evacuation orders: On 12 March, the 20-km–radius area around the plant was denoted as a “restricted area” with compulsory evacuation (Ministry of Economy Trade and Industry 2011). Subsequently, a 20- to 30-km zone became an “evacuation-prepared area in case of emergency,” and, because of the asymmetric deposition of radioactive fallout, a few villages in the 20- to 50-km zone were redesignated as “deliberate evacuation areas” (Prime Minister of Japan and his Cabinet 2011; Yoshida and Takahashi 2012). In order to mitigate the effects of internal contamination, radiological contaminant tests were implemented on 17 March to restrict the distribution of radiocontaminated foods (Ministry of Health Labour and Welfare 2011b). The government’s preparedness and response plans have been criticized for being inadequate and based on limited knowledge of nuclear disaster management: Many residents were not informed of the disaster until the evacuation day, and some were left with the entire decision-making responsibility without information needed to make wise decisions on evacuation (NAIIC 2012). A lack of clear guidelines confused residents about the best evacuation process, and also about when to return home. Thus, within the same city, residents’ individual evacuation history and behavior resulted in potentially different initial and chronic exposure periods as well as exposure mechanisms, giving researchers an opportunity to identify individuals at high risk of internal contamination and to provide lessons for the management of chronic internal contamination risk both in the affected areas in Fukushima and in the aftermath of future nuclear accidents.

There are currently no comprehensive assessments of risk factors for internal contamination after a major nuclear accident, and the available estimates are limited in scope and timeliness. The earliest published assessments of cesium contamination after the Chernobyl accident used data collected 1 year after the initial radiation release (Holmberg et al. 1988; Koch et al. 1992; Rabitsch et al. 1991) and were not conducted in populations from the areas closest to the accident. Assessments of residents of the areas closest to the Chernobyl plant were delayed for ≥ 4 years after the accident (Bernhardsson et al. 2011; DeVita et al. 2000; Takano 1994), and the period from 1986 to 1989 has been characterized as “information difficult to obtain” (Saenko et al. 2011). Most of the studies conducted after this period suffered from significant methodological flaws: Some did not assess risk factors for contamination directly, although some assessed levels of contamination in foods associated with the surveyed populations (Leppanen et al. 2011); most had small sample sizes; and some used forensic samples that contained no information about lifestyle habits or risk behaviors (Rabitsch et al. 1991). Despite these limitations, most of these studies identified high levels of internal cesium contamination even a year after the Chernobyl accident (Bernhardsson et al. 2011; Koch et al. 1992; Leppanen et al. 2011; Rabitsch et al. 1991), and many suggested inhalation of radionuclides deposited on external surfaces (Holmberg et al. 1988) and/or consumption of contaminated foods as the main risk factors for internal contamination (Anspaugh et al. 1988). Better understanding of these risk factors using data from a large community cohort surveyed as close as possible to the radiation release incident is necessary to develop better methods for internal contamination risk mitigation.

Because of this scarcity of studies conducted in the immediate aftermath of past disasters, a method for identifying individuals at high risk of internal contamination after a nuclear accident has not yet been defined (Hatch et al. 2005; Saenko et al. 2011; Upton 1981). Since October 2011, the Voluntary Internal Radiation Exposure Screening (VIRES) program has been conducted in Minamisoma, Fukushima, with the ultimate goal of monitoring the long-term health risks of the residents (Minamisoma Municipal General Hospital 2014). The present study expands on the initial report of baseline exposure prevalence (Tsubokura et al. 2012) using the data collected in the first year after the incident to investigate both the prevalence and levels of measurable internal contamination among the participants and to identify risk factors for the levels of internal contamination.

## Methods

*Study setting*. Minamisoma, a coastal city located 14–38 km north of the Fukushima Dai-ichi nuclear plant, is one of the most radioactively contaminated regions in Fukushima and is covered by all three evacuation zones (see Supplemental Material Figure S1) (NAIIC 2012; WHO 2012). Of 146,520 evacuees from Fukushima prefecture, 61,710 (42%) were from this city. A year after the disaster, even after the return of some residents, the population had dropped from its predisaster level of about 72,000 to < 43,000, with 32% being > 65 years of age (Maeda 2012; Statistics Bureau Director General for Policy Planning & Statistical Research and Training Institute 2010).

*Data collection*. From 1 October 2011 to 11 March 2012, the VIRES program was conducted free of charge for residents ≥ 6 years of age at the Minamisoma Municipal General Hospital. A program notification was sent to each household of the general population, including former residents who had evacuated elsewhere but were traced using the city’s family registry (Ministry of Justice 1962). All families officially resident in Japan are registered in such a family registry, which is used for public welfare and statistical purposes. Through collaboration with the Minamisoma municipal authorities, this registry could be used to distribute information about the screening program (Minamisoma City 2014). Because participation in this study was voluntary and based on self-referral, it was not possible to randomize, counter balance, or in any way adjust for any bias that would be induced by subject self-selection over time. The ultimate goal of the program is to continuously collect data of Minamisoma residents and to eventually monitor the long-term health consequences in the population. A Fastscan Model 2250 whole body counter (WBC; Canberra Inc., Northbrook, IL, USA), shielded against background radiation, was used to detect total body activity of radionuclides. Calibration of the machine was conducted regularly based on the method suggested by the company (Bronson et al. 1984).

With a 2-min scan, the detection limits were 210 Bq for ^134^Cs and 250 Bq for ^137^Cs. The WBC could only measure ^137^Cs and ^134^Cs and not ^131^I, because of its short half-life of 8 days, whereas the half-lives of ^137^Cs and ^134^Cs are 30 and 2 years, respectively (Christodouleas et al. 2011; Napier 2012; Yasunari et al. 2011). Measurements were made in total body activity (in becquerels) and concentration by body weight (in becquerels per kilogram).

A self-reported questionnaire was administered, and its contents were restructured in December 2011 to shift the focus from the risks associated with external exposure to risks associated with internal contamination: Questionnaire A (1 October to 14 December 2011; *n* = 4,045) asked about occupation (outdoor, indoor, unemployed, student, housewife, or other), hours spent outdoors in the first week of the disaster (< 5 hr or ≥ 5 hr), and changes in daily activity (diet and physical exercise). Questionnaire B (15 December 2011 to 11 March 2012; *n* = 4,236) asked questions about whether respondents selected certain food produce from the supermarket based on the possible radiocontamination risk at point of origin, or simply used local farms. It also asked whether respondents used bottled water when drinking and cooking, as opposed to tap or well water, to reflect government concerns about possible contamination of drinking water (Ministry of Health Labour and Welfare 2011a). Both questionnaires also asked the number of hours spent outdoors before and after the disaster as a proxy measure of external exposure to radiation (< 5 hr or ≥ 5 hr). These questionnaires were administered by the nurses and doctors managing the radiation assessment process and took only a few minutes to complete with the assistance of the attending staff. No member of the population answered both questionnaires A and B.

*Statistical analysis*. The median and range for basic radiation contamination data (total body contamination and concentration by body weight) are presented. We used chi-square tests to compare proportions. Throughout this article, we use the term “prevalence” to denote the proportion of individuals detected with internal contamination above the detection limit of the WBC machine.

We used Tobit regression analysis to determine risk factors for the levels of internal contamination. Tobit regression is an analysis method that adjusts standard linear regression models for the left-censoring effect of the WBC sensitivity, which creates a large proportion of zero values for participants with levels of contamination below the detection limit of the WBC. In conventional ordinary least squares regression, these participants would be treated as having zero contamination, when in fact they likely have some positive contamination at a level below the detection limit of the machine. Tobit regression adjusts for the effect of this lower limit on the model estimates. Because there were some outliers in the data, we conducted the Tobit regression analysis on the natural logarithm of the internal contamination data. All coefficients are in log-space and have been exponentiated in presenting regression results. This means that all the results represent multiplicative changes in internal contamination level corresponding to a unit change in the predictor. In order to model concentration (in becquerels per kilogram) rather than activity (in becquerels per body), we included the natural logarithm of weight as an offset in the Tobit model. This is equivalent to modeling the ratio of total body exposure and weight, but does not require the definition of a variable lower limit in the Tobit regression. The regression was adjusted for test month, sex, age, and height, and run on three data sets with slightly different sets of risk factor covariates: the whole sample and questionnaires A and B. Covariates were selected on the basis that previous studies of exposure risks after the Chernobyl accident identified the possibility that they would be related to either inhalation of airborne radionuclides or consumption of contaminated foods or water (Anspaugh et al. 1988). In addition, basic variables known to be associated with total body activity of internal concentration, such as height and age, were also included in the model selection process.

A variant backward-stepwise model-building method was used: First, a model was built for the overall data using a standard backward-stepwise method (Armitage et al. 2002) and a standard exclusion criterion of *p*-values > 0.05. All confounders identified in this model were then included in the data analysis on questionnaires A and B and retained in these models regardless of their significance. Confounders specific to questionnaires A and B, however, were excluded using the same standard backward-stepwise process. All the data were analyzed using Stata/MP, version 12 (StataCorp, College Station, TX, USA).

*Ethics and informed consent*. The institutional review board of the Institute of Medical Science, University of Tokyo, approved the study (code 23-46-0113), and informed consent was collected from all the participants before the study was conducted.

## Results

*Population characteristics*. A total of 8,829 individuals participated in the VIRES program during the study period (20% of the population a year after the disaster). Because of missing informed consent forms or questionnaire data, 548 individuals were excluded, leaving a complete data set of 8,281 individuals (94% of the initial enrollment).

By October 2011, approximately 43,000 residents (65% of the total registry) remained living in Minamisoma, whereas 6,400 participants of the program (77% of the data sample) answered that they stayed in the city (see Supplemental Material, Table S1. The registry only provides the data set with numbers rounded to the nearest 100). The data sample accounted for approximately 12% of the total city population under the registry. Comparison of age distributions between the data sample and the city’s family registry indicated that in both sexes there was an overrepresentation of those 10–19 years of age, and an underrepresentation of those > 60 years of age (see Supplemental Material, Figures S2 and S3). There was no significant difference in the number of women attending in the early period of the study (October–November) versus the later period (December–March) compared with men (χ^2^ = 1.39, *p* = 0.2).

The sample consisted of 7,214 adults [4,010 female; median age (range), 46 (16–94) years)] and 1,067 children [536 female; median age (range), 11 (6–15) years] (Table 1). After the accident > 87% of the respondents spent < 5 hr outdoors per day, compared with 80% before. During the early phase (1 October 2011 to 14 December 2011), > 70% of the participants answered that they were not careful about the origin of a food product when purchasing at the supermarket, and close to 50% were drinking tap water instead of bottled water (see Supplemental Material, Table S2). Later in the study (15 December 2011 to 11 March 2012), more than half were then paying extra attention to the origin of food products when purchasing them at the supermarket, and close to 70% were consuming non-tap water (see Supplemental Material, Table S3).

**Table 1:** Demographic characteristics.

Characteristic	*n* (%)
Type of questionnaire
Type A	4,045 (48.8)
Type B	4,236 (51.2)
Sex
Male	3,735 (45.1)
Female	4,546 (54.9)
Age category
Adult (> 15 years)	7,214 (87.1)
Child (≤ 15 years)	1,067 (12.9)
Age distribution (years)
0–9	292 (3.5)
10–19	1,747 (21.1)
20–29	658 (7.9)
30–39	1,239 (15.0)
40–49	1,176 (14.2)
50–59	1,205 (14.6)
60–69	1,133 (13.7)
70–79	620 (7.5)
≥ 80	211 (2.6)
Test month (2011–2012)^*a*^
October	1,412 (17.1)
November	1,739 (21.0)
December	1,777 (21.5)
January	1,831 (21.1)
February	1,388 (16.8)
March	134 (1.6)
Time spent outside before the disaster
< 5 hr/day	6,534 (79.4)
≥ 5 hr/day	1,697 (20.6)
Time spent outside after the disaster
< 5 hr/day	7,161 (86.5)
≥ 5 hr/day	1,120 (13.5)
Occupation^*b*^
Outdoor	632 (14.9)
Indoor	547 (12.9)
Unemployed	452 (10.7)
Student	456 (10.8)
Housewife	469 (11.1)
Other	1,422 (32.8)
Total sample	8,281 (100)
^***a***^Test month (2011–2012) refers to the number of participants who attended the screening program in each month. ^***b***^Data on occupation were collected only in questionnaire A.	

*Levels of internal radiation contamination*. A total of 2,969 individuals [2,874 adults (39.9% of the adult sample), 95 children (8.9% of the child sample)], 35.9% of the total sample, had some level of detectable internal contamination. The difference in the proportion of adults and children with levels of internal contamination above the detection limit of the WBC machine was statistically significant (χ^2^ = 396.72, *p* < 0.001). Similarly, differences in observed proportions were statistically significant by sex (χ^2^ = 771.58, *p* < 0.001) and by occupation (χ^2^ = 256.77, *p* < 0.001) (Table 2).

**Table 2:** Levels of internal contamination in cesium concentration by body weight.

Variable	*n*	Prevalence [*n* (%)]	Median (Bq/kg)^*a*^	Range (Bq/kg)^*a*^
Sex
Male	3,735	1,939 (51.9)	10.0	2.5–133.2
Female	4,546	1,030 (22.7)	12.2	2.3–196.5
Age category
Adult (> 15 years)	7,214	2,874 (39.9)	11.3	2.3–196.5
Child (≤ 15 years)	1,067	95 (8.9)	8.5	2.8–37.5
Occupation^*b*^
Outdoor	632	471 (74.5)	16.7	2.7–196.5
Indoor	547	244 (44.6)	10.2	2.6–137.9
Unemployed	452	275 (60.8)	14.1	2.4–93.4
Student	456	372 (81.6)	11.7	2.6–57.9
Housewife	469	160 (42.1)	10.5	2.8–53.1
Other	1,422	700 (49.2)	11.4	2.6–124.1
Total sample	8,281	2,969 (35.9)	11.3	2.3–196.5
^***a***^Individuals with measurements lower than the detection limits of WBC (i.e., < 250 Bq) were excluded from the calcu­la­tions of medians and ranges. ^***b***^Data on occupation were collected only in questionnaire A.				

**Figure 1:**
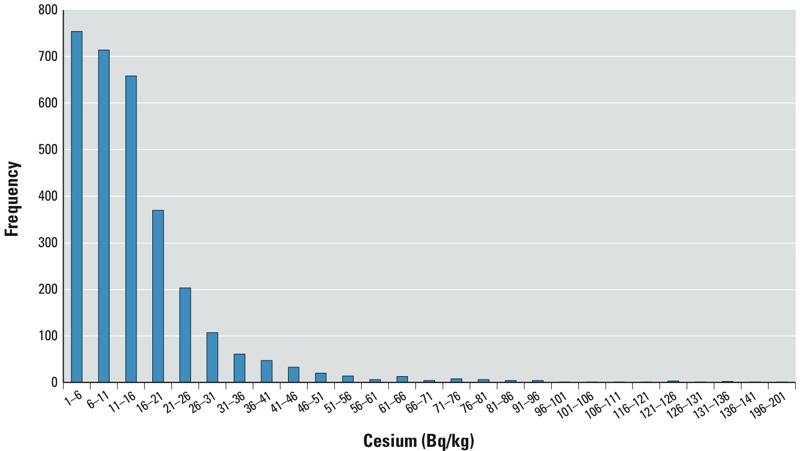
Histogram of cesium concentration in exposed individuals (*n* = 2,969).

The majority of individuals had contamination concentrations between 0 and 20 Bq/kg, and concentration > 50 Bq/kg was very rare (Figure 1). A clear declining trend in both monthly averaged concentration and detection rate during the study period was observed, with the detection rate < 1.6% in March 2012 (Table 1). The trend observed here is simply a monthly average of the detection rate, not a follow-up of the same individuals, and, because this is a cross-sectional study, the declining trend is not necessarily indicative of declining contamination levels within individuals.

*Risk factors for internal radiation contamination*. Overall, the levels of internal contamination gradually declined from October to March (*p* < 0.001; Table 3). Also the level was 59% lower in women than men (*p* < 0.001; Table 3), and 34% lower in children than in adults (*p* < 0.001; Table 3), suggesting contamination risk was lower in children even after adjusting for weight and the detection limit of the machine. Height and age were also risk factors for higher contamination, with older and/or taller individuals having higher levels of contamination (Table 3). Regardless of home address within Minamisoma, more time spent outdoors significantly increased the internal contamination concentration by 38% (*p* < 0.001; Table 3). These findings were consistent with the other findings listed in Table 4. For instance, outdoor workers had a higher contamination level when compared with indoor workers, housewives, and the unemployed (Table 4). The use of tap water and not taking precautions regarding the sources of foods (rice and vegetables) when purchasing from the supermarket were the second identified risk factor (attention to food and water intake) that significantly increased the contamination level (Table 5). The increased contamination risk for those who reported purchasing non-Fukushima–produced mushrooms from the supermarket was unexpected.

**Table 3:** Tobit multiple regression analysis on overall data (*n* = 8,281): relative change in detection rate.

Variable	Relative change (95% CI)^*a*^	SE	*t*-Statistic	*p*-Value
Test month (2011–2012)
>October	1 (Referent)
>November	0.38 (0.34, 0.42)	0.05	–20.07	< 0.001
>December	0.32 (0.29, 0.36)	0.05	–23.14	< 0.001
>January	0.23 (0.20, 0.25)	0.06	–26.90	< 0.001
>February	0.18 (0.16, 0.20)	0.06	–28.60	< 0.001
>March	0.15 (0.11, 0.21)	0.17	–11.20	< 0.001
Sex
>Male	1 (Referent)
>Female	0.41 (0.37, 0.44)	0.05	–19.94	< 0.001
Age category
>Adult (> 15 years)	1 (Referent)
>Child (≤ 15 years)	0.66 (0.55, 0.79)	0.09	–4.58	< 0.001
Height	1.72 (1.07, 2.77)	0.24	2.23	0.02
Age at test	1.21 (1.19, 1.24)	0.01	18.05	< 0.001
Time outside after disaster
>< 5 hr	1 (Referent)
>≥ 5 hr	1.38 (1.26, 1.51)	0.05	7.05	< 0.001
^***a***^Relative change in internal radiation contamination level.				

**Table 4:** Tobit multiple regression analysis on questionnaire A (*n* = 4,045): relative change in detection rate.

Variable	Relative change (95% CI)^*a*^	SE	*t*-Statistic	*p*-Value
Test month (2011–2012)
>October	1 (Referent)
>November	0.40 (0.37, 0.44)	0.05	–19.32	< 0.001
>December	0.36 (0.32, 0.40)	0.06	–17.92	< 0.001
Sex
>Male	1 (Referent)
>Female	0.45 (0.40, 0.51)	0.06	–13.28	< 0.001
Age category
>Adult (> 15 years)	1 (Referent)
>Child (≤ 15 years)	0.65 (0.50, 0.85)	0.13	–3.17	0.002
Height	1.34 (0.72, 2.49)	0.32	0.93	0.4
Age at test	1.18 (1.14, 1.22)	0.02	9.70	< 0.001
Time outside after disaster
>< 5 hr	1 (Referent)
>≥ 5 hr	1.28 (1.13, 1.45)	0.06	3.83	< 0.001
Occupation
>Outdoor	1 (Referent)
>Indoor	0.69 (0.60, 0.81)	0.08	–4.83	< 0.001
>Unemployed	0.68 (0.58, 0.79)	0.08	–4.97	< 0.001
>Student	0.81 (0.65, 1.01)	0.11	–1.89	0.06
>Housewife	0.60 (0.51, 0.71)	0.09	–5.94	< 0.001
>Other	0.72 (0.64, 0.81)	0.06	–5.53	< 0.001
Change in daily activity
>Hand wash and gargle	0.84 (0.77, 0.91)	0.04	–4.19	< 0.001
>Avoid tap water	0.76 (0.70, 0.82)	0.04	–6.47	< 0.001
>Wash vegetables	1.10 (1.01, 1.20)	0.04	2.25	0.03
^***a***^Relative change in internal radiation contamination level.				

**Table 5:** Tobit multiple regression analysis on questionnaire B (*n* = 4,236): relative change in detection rate.

Variable	Relative change (95% CI)^*a*^	SE	*t*-Statistic	*p*-Value
Test month (2011–2012)
>December	1 (Referent)
>January	0.73 (0.64, 0.83)	0.07	–4.61	< 0.001
>February	0.57 (0.49, 0.66)	0.07	–7.75	< 0.001
>March	0.49 (0.34, 0.70)	0.18	–3.93	< 0.001
Sex
>Male	1 (Referent)
>Female	0.39 (0.34, 0.46)	0.08	–12.28	< 0.001
Age category
>Adult (> 15 years)
>Child (≤ 15 years)	0.63 (0.48, 0.83)	0.14	–3.25	0.001
Height	2.37 (1.10, 5.11)	0.39	2.21	0.03
Age at test	1.24 (1.20, 1.28)	0.02	12.77	< 0.001
Time outside after disaster
>< 5 hr	1 (Referent)
>≥ 5 hr	1.30 (1.14, 1.48)	0.07	3.87	< 0.001
Attention to food and water intake^*b*^
>Rice	0.86 (0.76, 0.98)	0.06	–2.27	0.03
>Vegetables	0.66 (0.55, 0.78)	0.09	–4.73	< 0.001
>Mushrooms	1.25 (1.05, 1.49)	0.09	2.52	0.01
>Drinking water	0.87 (0.77, 0.98)	0.06	–2.29	0.02
^***a***^Relative change in internal radiation contamination level. ^***b***^Effects on overall internal contamination levels as a result of purchasing rice, vegetables, or mushrooms from outside of Fukushima prefecture, or of drinking only bottled water.				

## Discussion

The objective of this large-scale cross-sectional study was to assess the levels and risks of internal contamination after the Fukushima Dai-ichi nuclear accident. Minamisoma was the first municipality to adopt the WBC for internal contamination screening and also the first to provide it as a form of medical examination (Tsubokura et al. 2012). This study is also unique in its use of the WBC in combination with detailed evacuation history and behavioral data, which provided an opportunity to assess not only the levels of, but also risk factors for, internal contamination (NAIIC 2012; Yasumura et al. 2012).

The proportion of participants with detectable internal contamination was 40% in adults and 9% in children. Their levels of internal contamination ranged from 2.3 to 196.5 Bq/kg, and contamination > 50 Bq/kg was very rare. A year after the disaster, in March 2012, the overall detection rate had dropped from 60% to < 10% with the average cesium concentration < 2 Bq/kg. These substantial declines were more noticeable among children, possibly due to differences in their rates of metabolizing radioactive materials or to differences in risk behaviors (Hatch et al. 2005; Napier 2012). Such variation in overall contamination level among adults and children was also apparent in studies conducted after the Chernobyl disaster (Hatch et al. 2005). As opposed to findings from previous studies (Hatch et al. 2005), contamination risk by sex differed in this study, especially among adults, with a higher prevalence of internal contamination in males (52% vs. 22%; Table 2) but slightly higher concentration levels in women who were exposed (median level, 12 Bq/kg in women vs. 10 Bq/kg in men; Table 2). It is not clear whether such a difference is caused by biological or behavioral factors or is an artifact of the detection limit of the machine (Hatch et al. 2005). It is possible, for example, that women are in general more careful about exposure risks but that a small minority have higher exposure risks. We did not find women were more likely to attend the program earlier in the study, so this difference in contamination risk is unlikely to be due to selection bias induced by sex-related differences in WBC assessment time.

We identified two main risk factors for internal contamination: duration of time spent outdoors and intake of potentially contaminated foods and water. Paying more attention to the place of origin of foods, especially of vegetables and rice, significantly reduced contamination risk. Previous studies have also shown that contaminated groundwater, fruits, vegetables, and milk are the important risk factors for internal contamination (Hatch et al. 2005; Ministry of Health Labour and Welfare 2011a; Muck 1997; Napier 2012; Smith et al. 2000; Takatsuji et al. 2000). Mushrooms are also an important food product to consider, because they can accumulate a large amount of radionuclides through their slow decontamination process (Kalac 2001). In this study, selection of non-Fukushima–produced mushrooms significantly increased the contamination risk, contrary to expectations. This may be because respondents who can recall paying extra attention to mushroom preparation may have had very high levels of daily mushroom consumption. Although a reduction in contamination level observed due to food and water intake was substantial, and thus such behavioral measures may reflect the effectiveness of food control, this effect may also be a reflection of how careful an individual was after the disaster. For example, the purchase of non-Fukushima–produced rice was associated with contamination reduction, but rice on sale during the study was from the previous year’s harvest and could not have been radiologically contaminated. These variables may, therefore, reflect the respondents’ overall concerns rather than a specific benefit arising from consumption of specific products. Frequency of hand washing and gargling (two common Japanese personal hygiene practices) were also associated with reductions in the contamination risk but may be variables that reflect overall levels of concern about radiation and health, rather than direct risk factors for internal contamination.

The finding of low prevalence of and low levels of internal contamination was unexpected, considering the amount of radionuclides released, the duration of release, and the severity of soil contamination (WHO 2012; Yoshida and Takahashi 2012). Although we are not aware of malignancy among children or acute health problems, it is difficult to predict the future well-being of residents because an assessment of iodine contamination was not possible because of its short half-life and because the health consequences of low-dose radiation contamination are still unknown (Tsubokura et al. 2012; WHO 2013; Yasumura et al. 2012). In most of the affected areas [with estimated effective doses of 10–50mSv (NAIIC, 2012)], the increase in the relative lifetime risk of leukemia, breast cancer, all solid cancers, and thyroid cancer for an exposed infant would be approximately 7%, 6%, 4%, and 70%, respectively (WHO 2013). Nonetheless, even such a large increase in the relative risk becomes a small increase when converted to absolute risk (WHO 2013). Therefore, large-scale follow-up studies on internal contamination levels, such as the VIRES program, are vital to better understand the long-term effects of a nuclear disaster and to monitor the effect of decontamination and public health measures.

This study has several limitations. First and most importantly, the study was not implemented until > 200 days after the accident, and the biological half-life of cesium is only 100 days. This means that initial contamination levels had declined significantly by the time we began WBC measurements. Nonetheless, the VIRES program offers an important opportunity for early assessment of internal contamination risks. At 250 days after the accident, the internal contamination received through direct inhalation of the plume had declined to about 8% of its initial value, but with a detection limit of 210 Bq, our machine was still able to identify cesium contamination in individuals who had ingested at least 2,500 Bq during the accident. Experience in Chernobyl suggests that quantities much larger than this can be identified in initially exposed persons (WHO 2013). Negative results are also important in this context because, after more than three half-lives have elapsed, negative results can occur only if ongoing ingestion of cesium contaminants is not occurring—an important finding for those living within 30 km of a crippled nuclear power plant.

The WBC was not designed for use with children, which prevented accurate contamination assessment of those < 6 years of age. Our study is subject to selection bias toward the most highly concerned individuals with obvious risk factors (i.e., a bias toward the most health conscious in the community who exercise additional caution against radiation contamination even though they might be at low risk). This selection bias may partly explain the sudden drop in radiation contamination levels observed in November 2011, with limited further change in subsequent months (Table 3). Third, due to space constraints on the design of the questionnaires, detailed information about types of foods and water, and the precise amounts consumed, were not possible to obtain. Therefore, some of the questions were subjective, and should be seen as a proxy measure of the amount of foods and water consumed or potential for exposure to externally consumed radionuclides rather than precise measurements of this risk. Fourth, it was not possible to assess iodine contamination in this study because the short half-life of iodine meant that the exposure period for this isotope had largely passed by the time the VIRES program was begun. In addition to the lack of data on the level of internal ^131^I contamination, the relative proportions of ^131^I and cesium in the environment likely vary depending on the site in Minamisoma, so it was not possible to estimate the levels of iodine contamination in this study. This is unfortunate, because iodine was also the most poorly understood radiocontaminant released after the Chernobyl accident, but it was also the radiocontaminant most clearly linked to negative health effects (Saenko et al. 2011). The pattern observed after the Chernobyl accident is likely to be repeated in Minamisoma, but sadly the large contribution of ^131^I to the committed effective dose has been completely overlooked in the present study. Therefore, estimates of committed maximum radiation dose were underestimated due to absence of knowledge of this data. Nuclear disaster response plans need to be improved to ensure that iodine internal contamination estimates can be conducted in the immediate aftermath of the disaster and that effective monitoring can be implemented immediately, rather than after periods of months.

Another limitation of the present study is the method used for statistical analysis. Because the data are left-censored by the detection limit of the WBC machine, we used Tobit regression for statistical analysis; however, Tobit regression has several flaws that reduce its usefulness and validity. The concept of residuals is not well defined in Tobit regression, and methods for testing model validity were not available given the slightly unusual truncated nature of the conditional distribution of outcomes. The method also treats the detection limit as a fixed and non-stochastic limit, which is appropriate for many economic applications but may not be correct for radiological assessment equipment with an inherent machine error at this lower boundary. Commonly accessible implementations of Tobit regression also do not allow a varying left-detection limit, although this is defined in the original description of Tobit regression, and this required us to fit weight as an offset—a method that ultimately enabled us to estimate the effect of risk factors on activity (measured in becquerels per kilogram) but was not as good as modeling body activity directly. Finally, midway through the study, the questionnaire contents were changed because concerns about contamination risk shifted from immediate environmental sources of internal radiation contamination (such as inhalation of airborne particulates; questionnaire A) to avoidable ingestive sources of internal contamination (water and food; questionnaire B). Hence, external exposure risk assessment was only possible for the first 3 months and not for the entire year.

*Implications for disaster response*. The present study was conducted under challenging circumstances in a town experiencing rapid population changes after a devastating disaster that was responsible for the death of just over 1% of the population of the town. From our experience, we have been able to identify some key lessons for researchers and health care workers responding to such a nuclear accident under very difficult circumstances, some of which are detailed here.

*Maintain independence*. Independence bolsters the trust that the local community has in our work, and enables us to attract a wider range of individuals to the screening program, as well as ensuring that our results are believed and accepted when reported back to the local community.
*Provide feedback to the community*. One of the authors (M.T.) is involved in a regular program of radiation health seminars in Minamisoma, instructing residents on how to avoid further internal contamination. This program is informed by our research, informs us of the residents’ main risk behaviors, and enables our research to directly inform the community members who are involved in it. Through this community feedback, we can ensure that the program is sustainable and of benefit to the local community and identify new areas of research that may be necessary.
*Better preparation is essential*. Communities located near nuclear power plants need to be prepared for the worst-case scenario, and a part of that preparation should include the presence of a WBC machine, or the identification of a machine, that can be rapidly deployed. Our results would have had much more power if we had been able to begin the VIRES program within 100 days of the accident, but this was impossible simply because of a lack of local preparation.
*Ensure rapid assessment.* The biological half-life of cesium is just 100 days, and that of iodine even shorter. In addition to timely implementation of assessment, it is essential to be able to rapidly process large numbers of residents in a short time in order to ensure that the assessment process does not unduly restrict the sample size that can be gathered in the first days of the program when internal contamination levels will be declining most rapidly. We designed a one-page questionnaire to be conducted in conjunction with a 2-min scan, and we conducted no blood tests or other health tests. This ensured that we could process large numbers of people rapidly, scanning 3,000 individuals in just 2 months with only one machine and a couple of staff members. A slower assessment process would significantly reduce the available sample size in the early period of the study when detection is most likely, reducing the ability of the study to effectively assess committed dose or distinguish between acute and chronic exposure.

With the lessons learned from this study, we hope that communities that live near nuclear power plants will be better able to respond to such accidents in future, with all the benefits for local population health that this rapid response implies.

## Conclusion

A nuclear disaster has multiple effects on health: directly, by internal contamination; indirectly, by psychological disturbance such as anxiety about unforeseen future and social disruption; or indirectly, through changes in lifestyle associated with behavioral changes required to reduce external exposure (Sugimoto et al. 2012; Wind et al. 2011; Yasumura et al. 2012). Unless the latter two effects are properly managed, they may be more harmful to individuals than the effects of the radiation itself (Sugimoto et al. 2012). Past practices in nuclear disaster management have predominantly involved evacuation and relocation, but recent studies from Chernobyl suggest that weak regulation of foods and water was the main cause of the prolonged internal contamination effect and of increased cancer incidence (Hatch et al. 2005; Saenko et al. 2011). A standard for the minimum airborne radiation level for acute exposure should be clearly noted, but the level of internal contamination is uncorrelated with either external radiation level or distance to the plant. This is because high levels of airborne radionuclides only persist during the initial stages (Muck 1997). Thus, to mitigate the overall effects, the benefits of initial evacuation must be weighed against the long-term social disruption and health consequences of extended evacuation; and where evacuation is considered too dangerous or disruptive, rigorous ongoing monitoring of radiation contamination should be implemented early and maintained throughout the affected communities.

An alternative form of nuclear disaster response plan should include all of the following (Napier 2012; Rahu 2003; ten Hoeve and Jacobson 2012:

Careful assessment of the necessity of long-term evacuation, with the ultimate focus on the risk profile of individual residents.
Strict enforcement of food and water control, with the immediate commencement of radiological contaminant tests and sales restrictions.
Dissemination of evidence-based, clear, and up-to-date information on what is known and unknown about methods to reduce internal contamination, for self-protection and to diminish concerns of affected individuals.

Although the developed world is shifting toward an anti-nuclear movement, developing nations are in need of nuclear power to support their industrialization. Unless the use of nuclear power is abolished, the chance of another nuclear accident remains. Because nuclear disasters are rare, it is often the case that communities are neither experienced nor prepared for their aftermath (Christodouleas et al. 2011). Our research suggests that radiation contamination risks can be identified and may be managed and that, with sensible and reasonable precautions, people may be able to continue to live in radiation-affected areas with limited risk of contamination. However, it is imperative that governments in nations with nuclear industries be aware of the worst-case scenarios, develop a rational and evidence-based emergency response, and be prepared for both the short- and long-term effects of radiation contamination.

## Supplemental Material

(346 KB) PDF

## References

[r1] Anspaugh L, Catlin R, Goldman M (1988). The global impact of the Chernobyl reactor accident.. Science.

[r2] Armitage P, Berry G, Matthews JNS. (2002). Statistical Methods in Medical Research.

[r3] Bernhardsson C, Zvonova I, Rääf C, Mattsson S (2011). Measurements of long-term external and internal radiation exposure of inhabitants of some villages of the Bryansk region of Russia after the Chernobyl accident.. Sci Total Environ.

[r4] Bronson F, Booth L, Richards D. (1984). Fastscan: A Computerized, Anthropometrically Designed, High Throughput, Whole Body Counter for the Nuclear Industry. Northbrook, IL:Canberra Industries Inc.. http://www.canberra.com/literature/invivo_counting/tech_papers/fastscan.pdf.

[r5] Christodouleas JP, Forrest RD, Ainsley CG, Tochner Z, Hahn SM, Glatstein E (2011). Short-term and long-term health risks of nuclear-power-plant accidents.. N Engl J Med.

[r6] DeVita R, Olivieri A, Spinelli A, Grollino MG, Padovani L, Tarroni G (2000). Health status and internal radiocontamination assessment in children exposed to the fallout of the Chernobyl accident.. Arch Environ Health.

[r7] Hatch M, Ron E, Bouville A, Zablotska L, Howe G (2005). The Chernobyl disaster: cancer following the accident at the Chernobyl nuclear power plant.. Epidemiol Rev.

[r8] Holmberg M, Edvarson K, Finck R (1988). Radiation doses in Sweden resulting from the Chernobyl fallout: a review.. Int J Radiat Biol.

[r9] Kalac P (2001). A review of edible mushroom radioactivity.. Food Chem.

[r10] Koch HC, Burmeister W, Knopp R, Niesen M, Georgakopoulou A, Kramer A (1992). Whole-body cesium 137 activity up to 4 years after the Chernobyl reactor accident in premature newborns, newborns, infants, and children.. Pediatrics.

[r11] Leppanen AP, Muikku M, Jaakkola T, Lehto J, Rahola T, Rissanen K (2011). Effective half-lives of ^134^Cs and ^137^Cs in reindeer meat and in reindeer herders in Finland after the Chernobyl accident and the ensuing effective radiation doses to humans.. Health Phys.

[r12] Maeda K. (2012). Mental Health in Minamisoma City, Fukushima [in Japanese].. http://184.73.219.23/rounen/news/houkoku201209.htm.

[r13] Minamisoma City. (2014). Mimamisoma City Homepage [in Japanese].. http://www.city.minamisoma.lg.jp/index.cfm/1,html.

[r14] Minamisoma Municipal General Hospital. (2014). Minamisoma Municipal General Hospital Homepage [in Japanese].. http://sogohp.or.jp/.

[r15] Ministry of Economy Trade and Industry. (2011). Setting of the Planned Evacuation Zone and Evacuation Prepared-Area in Case of Emergency.. http://www.atomdb.jnes.go.jp/content/000118461.pdf.

[r16] Ministry of Health Labour and Welfare. (2011a). Detection of Radioactive Materials in Tap Water in Fukushima Prefecture (March 22).. http://www.mhlw.go.jp/english/topics/2011eq/dl/march_22_01.pdf.

[r17] Ministry of Health Labour and Welfare. (2011b). Handling of Food Contaminated by Radioactivity (relating to the Accident at the Fukushima Nuclear Power Plant).. http://www.mhlw.go.jp/stf/houdou/2r9852000001558e-img/2r98520000015apy.pdf.

[r18] Ministry of Justice. (1962). Laws and Regulations relating to Court Proceedings for Family Affairs and Family Registration.

[r19] Muck K (1997). Long-term effective decrease of cesium concentration in foodstuffs after nuclear fallout.. Health Phys.

[r20] NAIIC (The National Diet of Japan Fukushima Nuclear Accident Independent Investigation Commission). (2012). Chapter 4. Overview of damage from the nuclear power plant accident. In: The National Diet of Japan Fukushima Nuclear Accident Independent Investigation Commission.. http://warp.da.ndl.go.jp/info:ndljp/pid/3856371/naiic.go.jp/wp-content/uploads/2012/08/NAIIC_Eng_Chapter4_web.pdf.

[r21] Napier B (2012). Estimation of internal radiation dose from both immediate releases and continued exposures to contaminated materials.. J Radiol Prot.

[r22] Nuclear Energy Agency, Organisation for Economic Co-Operation and Development. (2002). Chernobyl: Assessment of Radiological and Health Impacts. 2002 Update of Chernobyl: Ten Years On.. http://www.oecd-nea.org/rp/reports/2003/nea3508-chernobyl.pdf.

[r23] Prime Minister of Japan and his Cabinet. (2011). Selection of “Planned Evacuation Zone” and “Evacuation Zone in Case of Emergency” [in Japanese].. http://www.kantei.go.jp/saigai/pdf/201104220944siji.pdf.

[r24] Rabitsch H, Feenstra O, Kahr G (1991). Radiocesium levels in humans over a four-year period.. J Nucl Med.

[r25] Rahu M (2003). Health effects of the Chernobyl accident: Fears, rumours and the truth.. Eur J Cancer.

[r26] Saenko V, Ivanov V, Tsyb A, Bogdanova T, Tronko M, Demidchik Y (2011). The Chernobyl accident and its consequences.. Clin Oncol (R Coll Radiol).

[r27] Smith JT Comans RN Beresford NA Wright SM Howard BJ Camplin WC 2000 Chernobyl’s legacy in food and water. Nature 405 141; 10.1038/35012139 10821261

[r28] Statistics Bureau Director General for Policy Planning & Statistical Research and Training Institute. (2010). Report Chapter 2. Housing Condition in Minamisoma City, Fukushima.. http://www.city.minamisoma.lg.jp/mpsdata/web/3092/js-02.pdf.

[r29] Sugimoto A, Krull S, Nomura S, Morita T, Tsubokura M (2012). The voice of the most vulnerable: lessons from the nuclear crisis in Fukushima, Japan.. Bull World Health Organ.

[r30] Taira Y Hayashida N Tsuchiya R Yamaguchi H Takahashi J Kazlovsky A 2013 Vertical distribution and estimated doses from artificial radionuclides in soil samples around the Chernobyl nuclear power plant and the Semipalatinsk nuclear testing site. Plos One 8 e57524; 10.1371/journal.pone.0057524 23469013 PMC3585370

[r31] Takano K 1994 Cesium-137 residues in food and in persons in areas severely contaminated by the Chernobyl power station accident [in Japanese] Nihon Koshu Eisei Zasshi [Japanese Journal of Public Health] 41 920 925 7949290

[r32] Takatsuji T, Sato H, Takada J, Endo S, Hoshi M, Sharifov VF (2000). Relationship between the ^137^Cs whole-body counting results and soil and food contamination in farms near Chernobyl.. Health Phys.

[r33] ten Hoeve JE, Jacobson MZ (2012). Worldwide health effects of the Fukushima Daiichi nuclear accident.. Energy Environ Sci.

[r34] Tokyo Electronic Power Company. (2012). An Estimate of Radioactive Substances Released into the Atomosphere after the Fukushima Dai-Ichi Nuclear Accident [in Japanese].. http://www.tepco.co.jp/cc/press/betu12_j/images/120524j0101.pdf.

[r35] Tsubokura M, Gilmour S, Takahashi K, Oikawa T, Kanazawa Y (2012). Internal radiation exposure after the Fukushima nuclear power plant disaster.. JAMA.

[r36] Upton AC (1981). Health impact of the Three Mile Island accident.. Ann NY Acad Sci.

[r37] WHO (World Health Organization). (2012). Preliminary Dose Estimation from the Nuclear Accident after the 2011 Great East Japan Earthquake and Tsunami.. http://whqlibdoc.who.int/publications/2012/9789241503662_eng.pdf.

[r38] WHO (World Health Organization). (2013). Health Risk Assessment from the Nuclear Accident after the 2011 Great East Japan Earthquake and Tsunami—Based on a Preliminary Dose Estimation.. http://apps.who.int/iris/bitstream/10665/78218/1/9789241505130_eng.pdf.

[r39] Wind TR Fordham M Komproe IH 2011 Social capital and post-disaster mental health. Glob Health Action 4; 10.3402/gha.v4i0.6351 PMC3118777 21695072

[r40] Yasumura S, Hosoya M, Yamashita S, Kamiya K, Abe M, Akashi M (2012). Study protocol for the Fukushima Health Management Survey.. J Epidemiol.

[r41] Yasunari TJ, Stohl A, Hayano RS, Burkhart JF, Eckhardt S, Yasunari T (2011). Cesium-137 deposition and contamination of Japanese soils due to the Fukushima nuclear accident.. Proc Natl Acad Sci USA.

[r42] Yoshida N, Takahashi Y (2012). Land-surface contamination by radionuclides from the Fukushima Daiichi nuclear power plant accident.. Elements.

