# Supplementary material for: Assessment of the Risk of Medium-Term Internal Contamination in Minamisoma City, Fukushima, Japan, after the Fukushima Dai-ichi Nuclear Accident
Source: Environ Health Perspect. 2014 Mar 14;122(6):587–93. doi: 10.1289/ehp.1306848 (PMC4050509; doi:10.1289/ehp.1306848)
Supplement: (346 KB) PDF [file ehp.1306848.s001.pdf]

Environmental Health Perspectives doi:10.1289/ehp.1306848

Supplemental Material

Assessment of the Risk of Medium-Term Internal Contamination in Minamisoma City, Fukushima, Japan, after the Fukushima Dai-ichi Nuclear Accident

Amina Sugimoto, Stuart Gilmour, Masaharu Tsubokura, Shuhei Nomura, Masahiro Kami, Tomoyoshi Oikawa, Yukio Kanazawa, and Kenji Shibuya

Table of Contents **Page**

Figure S1: Map of Fukushima with three distinct evacuation zones 2

Table S1: Proportion of Minamisoma residents who left or stayed in the city compared with study respondents 3

Figure S2: Comparison of age distribution between the sample and the Minamisoma family registry (male) 4

Figure S3: Comparison of age distribution between the sample and the Minamisoma family registry (female) 5

Table S2: Summary of risk factors by age categories (Questionnaire A: n=4,045) 6

Table S3: Summary of risk factors by age categories (Questionnaire B: n=4,236) 7

Table S4: Summary of external exposure 8

Table S5: Evacuation history summary 9

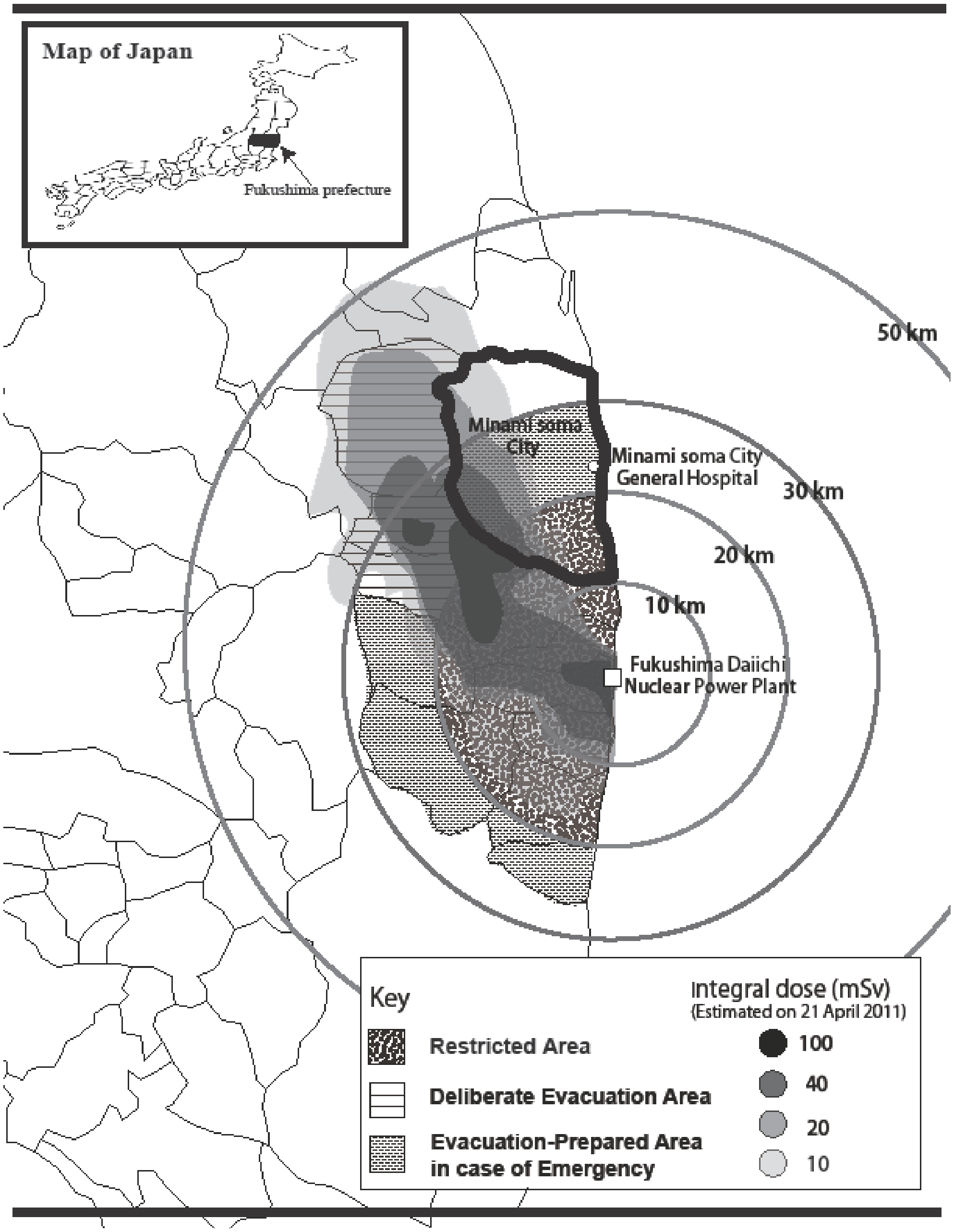

Figure S1: Map of Fukushima with three distinct evacuation zones.

(As of May 2011, within the 20km zone was an entry restricted area, the majority of areas within the 30 km zone were classified as the deliberate evacuation areas, and parts of northern Fukushima within the 50km zone were defined as evacuation-prepared areas in case of Emergency. Minamisoma city covered all three of the evacuation zones).

Table S1: Proportion of Minamisoma residents who left or stayed in the city compared with study respondents.

Group	Left Minamisoma: Number	Left Minamisoma: Percent	Stayed in Minamisoma: Number	Stayed in Minamisoma: Percent	Total
Family registry (October 2011)	23000	35	43000	65	66000
Sample	1900	23	6400	77	8300

*Note: All the values are rounded to nearest hundred, due to a lack of precise data in the city's family registry and frequent mobility of the residents at the time.

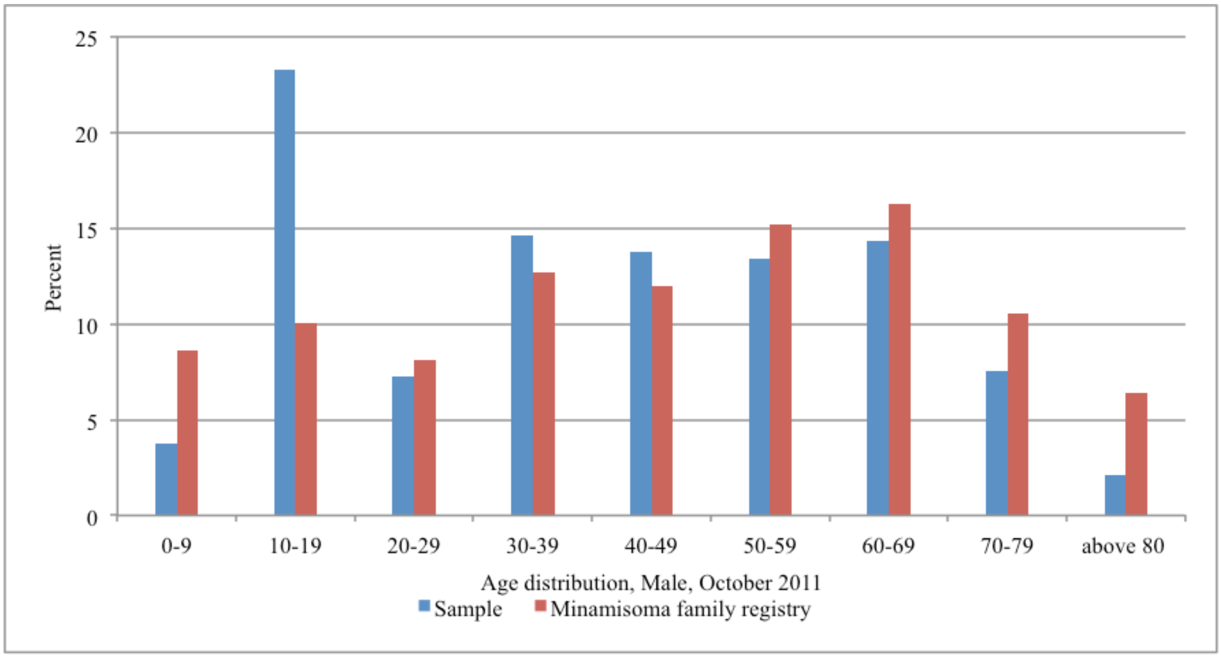

Figure S2: Comparison of age distribution between the sample and the Minamisoma family registry (male).

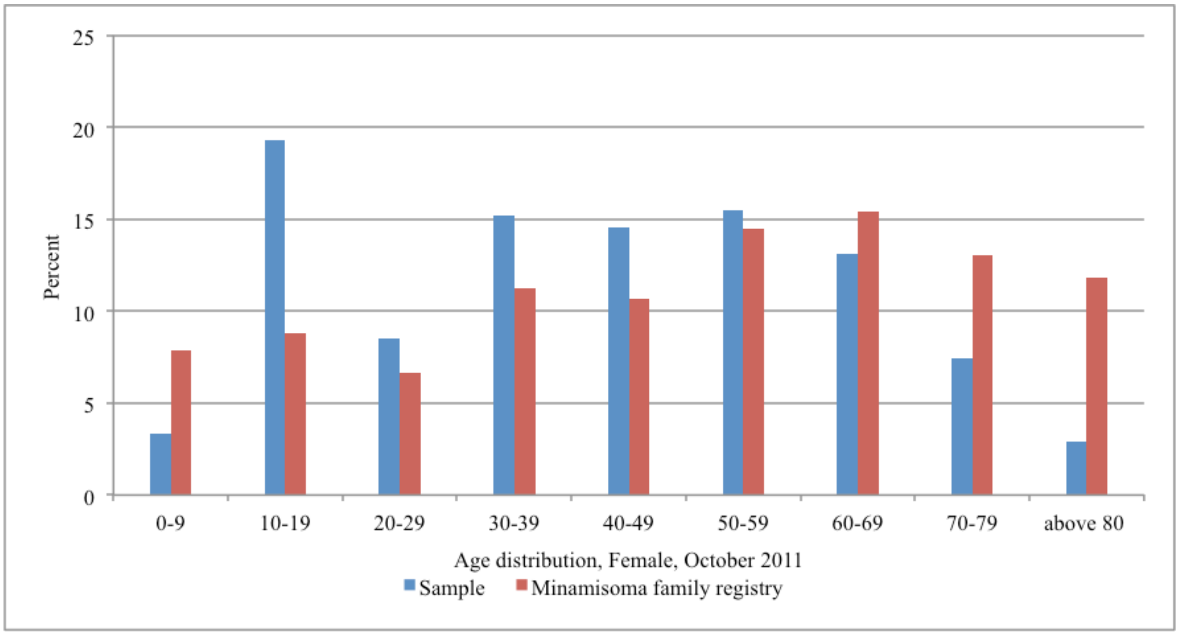

Figure S3: Comparison of age distribution between the sample and the Minamisoma family registry (female).

Table S2: Summary of risk factors by age categories (Questionnaire A: n=4,045).

Variables	Adults: N	Adults: %	Children: N	Children: %	P value
**Wear a mask**					
No	2158	56.4%	91	41.7%	
Yes	1669	43.6%	127	58.3%	<0.001
**Hand wash/gargle**					
No	1712	44.7%	80	36.7%	
Yes	2115	55.3%	138	63.3%	0.02
**Avoid certain foods**					
No	2778	72.6%	178	81.7%	
Yes	1049	27.4%	40	18.3%	0.003
**Avoid tap water**					
No	2351	58.8%	112	51.4%	
Yes	1576	41.2%	106	48.6%	0.03
**Wash vegetables**					
No	2082	54.4%	117	53.7%	
Yes	1745	45.6%	101	46.3%	0.8

Table S3: Summary of risk factors by age categories (Questionnaire B: n=4,236).

("Select by location" or "Select irrespective of location" refer to the choice people make when purchasing food products at the supermarket).

Variable	Adults: N	Adults: %	Children: N	Children: %	P value
**Rice**					
Select by location	1716	50.7%	482	56.8%	
Select irrespective of location	1671	49.3%	367	43.2%	0.001
**Meat**					
Select by location	2389	69.7%	626	73.7%	
Select irrespective of location	1028	30.3%	223	26.3%	0.02
**Fish**					
Select by location	2393	70.7%	656	77.3%	
Select irrespective of location	994	29.3%	193	22.7%	<0.001
**Vegetables**					
Select by location	2278	67.3%	646	76.1%	
Select irrespective of location	1109	32.7%	203	23.9%	<0.001
**Mushrooms**					
Select by location	2449	72.3%	664	78.2%	
Select irrespective of location	938	27.7%	185	21.8%	<0.001
**Milk**					
Select by location	2222	65.6%	616	72.6%	
Select irrespective of location	1165	34.4%	233	27.4%	<0.001
**Drinking Water**					
Tap water / Well	1219	36.0%	147	17.3%	
Other (e.g. bottled)	2168	64.0%	702	82.7%	<0.001
**Cooking water**					
Tap water / Well	549	16.2%	57	6.7%	
Other (e.g. bottled)	2838	83.8%	792	93.3%	<0.001

Table S4: Summary of external exposure.

**Variable**	**Median**	**Minimum**	**Maximum**
**Estimated average external exposure** [a]			
Total exposure [mSv/day]	3.2	0	12.3
Initial exposure (March) [mSv/day]	3.2	0	12.3
Prolonged exposure (April-December) [mSv/day]	0	0	4.5

[a]For simplicity, external exposures listed here are expressed in mSv/day. For multiple regression models, total exposure values in mSv were used.

Table S5: Evacuation history summary.

Variable	Median	Minimum	Maximum
**Evacuation history**			
Days spent in Fukushima	7	0	367
Days spent in Fukushima (March)	3	0	21
Days spent in Fukushima (April–December)	0	0	346
